# Evolving role of seneca valley virus and its biomarker TEM8/ANTXR1 in cancer therapeutics

**DOI:** 10.3389/fmolb.2022.930207

**Published:** 2022-08-26

**Authors:** Virginia Corbett, Paul Hallenbeck, Piotr Rychahou, Aman Chauhan

**Affiliations:** ^1^ Department of Internal Medicine, Icahn School of Medicine at Mount Sinai, New York, NY, United States; ^2^ Seneca Therapeutics, Inc., Blue Bell, PA, United States; ^3^ Department of Surgery, Markey Cancer Center, University of Kentucky, Lexington, KY, United States; ^4^ Division of Medical Oncology, Department of Internal Medicine, Markey Cancer Center, University of Kentucky, Lexington, KY, United States

**Keywords:** seneca valley virus, oncolytic virus, drug development, TEM8/ANTXR1, neuroendocrine tumors, neuroendocrine carcinomas, solid tumors

## Abstract

Oncolytic viruses have made a significant inroad in cancer drug development. Numerous clinical trials are currently investigating oncolytic viruses both as single agents or in combination with various immunomodulators. Oncolytic viruses (OV) are an integral pillar of immuno-oncology and hold potential for not only delivering durable anti-tumor responses but also converting “cold” tumors to “hot” tumors. In this review we will discuss one such promising oncolytic virus called Seneca Valley Virus (SVV-001) and its therapeutic implications. SVV development has seen seismic evolution over the past decade and now boasts of being the only OV with a practically applicable biomarker for viral tropism. We discuss relevant preclinical and clinical data involving SVV and how bio-selecting for TEM8/ANTXR1, a negative tumor prognosticator can lead to first of its kind biomarker driven oncolytic viral cancer therapy.

## Introduction: The promise of oncolytic viruses in cancer therapeutics

Immunotherapy has revolutionized the cancer treatment landscape. The development of immune checkpoint inhibitors targeting PD-1/PD-L1 or CTLA4 has improved patient outcomes in a variety of solid tumors (Kraehenbuehl et al., 2022). Chimeric antigen receptor T cells (CAR-T) and bispecific antibodies (bsAbs)/bispecific T-cell engagers (BiTEs) well developed in hematologic malignancies, are now being advanced in solid tumors (Edeline et al., 2021). Ongoing studies are evaluating cancer vaccines as well as a variety of combination therapies. Oncolytic viruses (OVs) represent an exciting and rapidly evolving field within cancer immunotherapies. Interest in using viruses in cancer treatment has been present for many years based on observations that many hematological malignancies temporarily improved with concurrent viral infections (Kelly and Russell, 2007). Recently, interest in OVs and OV combination therapies has surged, both with new insights into immunology and with rapid improvements in techniques for genetic engineering of viruses. Talimogene laherparepvec (T-VEC or trade name IMLYGIC™) is an OV based on a modified herpes simplex virus (HSV) type 1 with the addition of a gene encoding human granulocyte macrophage colony-stimulating factor (GM-CSF). The FDA approval of intratumoral injection of T-VEC in advanced melanoma in 2015 was the first in class approval of an oncolytic viral agent and has generated interest in additional trials evaluating OVs and novel OV combinations (Andtbacka et al., 2015; Zhang and Rabkin, 2021).

OV immunotherapy employs viruses that target cancer cells, either due to inherent characteristics of the virus or engineering for tumor selectivity. The primary mechanism of action includes two potential pathways 1) selective, replication in, and direct lytic destruction of tumor cells *in situ* and 2) induction of systemic anti-tumor immunity (Kaufman et al., 2015). The specific mechanism of action varies depending upon the viral vector, specific cancer cell type, addition of immune stimulatory agents, and modulation of the tumor microenvironment. Greater than 30 viruses have been evaluated in this setting including herpesvirus, adenovirus, poxvirus, picornavirus, reovirus among others (Cook and Chauhan, 2020). Recombinant engineering allowing enhancement of viral selectivity and response and/or removal of virulence genes has led to the creation of targeted and safe OVs (Boagni et al., 2021). Although direct destruction of tumor cells is key to the mechanism of OVs, recent studies suggest that the immune induction likely plays a more important role in their efficacy (Ramelyte et al., 2021). As OVs target and induce lysis of tumor cells, antiviral signals are triggered in the cells leading to endoplasmic reticulum stress and generation of antiviral cytokines and type I interferons (IFNs) which activate immune cells including antigen presenting cells and cytotoxic CD8^+^ T cells (Workenhe and Mossman, 2014). As the tumor is destroyed danger-associated molecular patterns (DAMPs) and pathogen-associated molecular patterns (PAMPs) are released further prompting an adaptive immune response by activation of toll like receptors (TLRs) (Malogolovkin et al., 2021). Tumor-associated antigens and neoantigens released by the dying cells cultivate tumor antigen-specific CD4^+^ and CD8^+^ T cell responses (Workenhe and Mossman, 2014; Kaufman et al., 2015). However, while OVs may stimulate an anti-tumor immune response this mechanism may also lead to an immune response against the OV including the production of neutralizing antibodies. The balance of anti-tumor and antiviral effects represent an important mediator of the efficacy of OVs (Grillo et al., 2018; Zhang and Rabkin, 2021). Delivery of OV by intratumoral injection in many cases seems to thwart neutralizing antibody inactivation. However, efficient, multiple intravenous administration is still an important goal in bringing this technology to more patients with varying solid cancers and in creating more tolerable therapies.

## Seneca valley virus targets neuroendocrine cancers

Seneca valley virus (SVV-001) is a naturally occurring oncolytic picornavirus first discovered in 2002 in a cell culture presumably contaminated with SVV-001 containing porcine trypsin or bovine serum. Soon after discovery, it was found to have selectivity for tumor cells with neuroendocrine properties (Reddy et al., 2007). SVV-001 is a single positive stranded, non-recombinant RNA virus (27 nm) that causes cell death *via* intracellular viral replication, cell lysis, and autophagy, with a replication cycle less than 12 h (Rudin et al., 2011; Burke, 2016). Complete genome sequencing revealed SVV-001 is a picornavirus, within a separate genus now called Senecavirus, closely related to cardioviruses (Venkataraman et al., 2008a; Venkataraman et al., 2008b; Hales et al., 2008). Of note, as an RNA virus there is no chance of insertion into the host genome and no risk of mutagenesis and SVV-001 was recognized soon after discovery as a promising candidate for OV therapy.

Most humans do not have antibodies to SVV-001 and normal, healthy human cells are not infected by SVV-001 (Molecular Theraphy, 2005). In contrast to other oncolytic viral agents under investigation, SVV-001 is not inhibited by normal human blood components (Reddy et al., 2007). The family of Seneca viruses has since been renamed Seneca virus A (SVA). SVA strains have been classified into 3 distinct clades. SVV-001, the original isolate from 2002 is in clade 1 of the Senecavirus genus. This agent, particularly when produced on the human cell line PER.C6 appears to be non-pathogenic in humans and swine and likely most or all animals (Fernandes et al., 2018). SVA in clades 2 and 3 are causative agents for vesicular disease in pigs (Jayawardena et al., 2019). SVV-001 has several unique features that make it attractive as an OV including: 1) potential targeting of solid tumors with intravenous dosing, 2) RNA virus without insertional mutagenesis, 3) *in vivo* self-replication.

When first identified SVV-001 was found to infect and replicate in cells with neuroendocrine markers, including gastrin releasing peptide receptors, synaptophysin, neuron specific enolase, and CD56 (Reddy et al., 2007; Bolton et al., 2020). However, in the last decade our understanding of the mechanism of specificity of SVV-001 for neuroendocrine cells has rapidly expanded. The tropism of SVV-001 for specific neuroendocrine tumors was explored in a study of SVV-001 in non-permissive small cell lung cancer (SCLC) cell lines. The authors identified a subpopulation of cells infected with SVV-001 in a model of SCLC previously thought to be resistant to infection (Poirier et al., 2012). This is likely due to targeting of cancer stem cells, which in a medulloblastoma orthotopic xenograft mouse model were found to be preferentially targeted by SVV-001 (Yu et al., 2011). Further work seeking to identify markers of infectivity to SVV-001 was done using a mouse model of SCLC. In this study 2 out of 6 mice exposed to a SVV-001 had durable, complete responses to therapy. Gene profiling was done of responders and compared to non-responders. Response to SVV-001 was correlated with a high expression of the transcriptomic regulator neurogenic differentiation factor 1 (*NEUROD1*) and low expression of achaete-scute homologue 1 (*ASCL1*) (Poirier et al., 2013). Of historical interest the tropism of SVV-001 for SCLC cells with low *ASCL1* to *NEUROD1* ratio was one of the initial observations that prompted further investigation into novel subtypes of SCLC, classified by expression of master transcriptomic regulators that are emerging as an important area of investigation and biomarkers of response to treatment. In the classification described by Rudin et al. (2019), SCLC with a low *ASCL1* to *NEUROD1* ratio is labeled as SCLC-N (Gay et al., 2021) (Table 1).

**TABLE 1:** Summary of key preclinical studies of SVV-001 in cancer cell lines and murine models.

References	Study/model	Outcomes
Reddy et al. (2007)	• Cytotoxicity evaluation in multiple tumor cell lines with neuroendocrine properties	• Most cell lines with neuroendocrine markers were sensitive to SVV-001 mediated killing, normal human cells were resistant to SVV-001 mediated killing
	• Toxicity evaluation in immunocompetent mice	• Toxicity evaluation in immunocompetent mice without dose limiting toxicity. Neutralizing antibodies were noted
	• Efficacy evaluation in athymic female tumor bearing mice with tumors derived from SCLC and retinoblastoma cell lines	• Efficacy analysis in athymic mice with promising anti-tumor killing efficacy in a model of tumors derived from SCLC and retinoblastoma cell lines
Wadhwa et al. (2007)	• Cytotoxicity evaluation in multiple tumor cell lines with neuroendocrine properties, including retinoblastoma, glioblastoma, and human embryonic kidney	• Cytotoxicity was noted with SVV-001 treatment in retinoblastoma cell lines but not glioblastoma or embryonic kidney cell lines
	• Efficacy evaluation in murine xenograft model of metastatic retinoblastoma created with injection of human retinoblastoma tumor cells into vitreous	• In the murine xenograft model of metastatic retinoblastoma intravenous administration of SVV-001 decreased extraocular tumor burden and decreased extraocular extension of tumor as compared to controls
Morton et al. (2010)	• Cytotoxicity of SVV-001 evaluated in 23 cancer cell lines	• Cytotoxicity noted with SVV-001 treatment in cell lines from a subset of neuroblastoma, Ewing sarcoma, and rhabdomyosarcoma panels
	• Efficacy evaluation in 36 solid tumor xenograft severe combined immunodeficiency (SCID) murine models	• In solid tumor xenograft murine models of rhabdomyosarcoma and neuroblastoma complete responses were observed with intravenous SVV-001 treatment, responses were also noted in rhabdoid tumor, Wilms tumor, and glioblastoma models
Yu et al. (2011)	• Efficacy evaluation of intravenous SVV-001 in a medulloblastoma orthotopic xenograft Rag2 SCID murine model	• Intravenous SVV-001 injection was associated with anti-tumor activity in medulloblastoma xenograft murine models and prolonged survival
		• Intravenous SVV-001 injection was associated with killing of cancer stem cells
		• SVV-001 treatment was associated with autophagy activation
		• SVV-001 was shown to cross the blood brain barrier *in vivo*
Poirier et al. (2013)	• Efficacy evaluation of intravenous SVV-001 in several classic and variant SCLC heterotransplant models immunosuppressed mice	• Efficacy was noted with tumor inhibition in variant SCLC heterotransplant models
	• Analysis of gene expression profiles in SVV-001 permissive tumors as compared to SVV-001 non-permissive tumors	• SVV-001 permissive tumors were associated with a specific gene profile characterized by elevated NEUROD1 to ASCL1 ratio
Miles et al. (2017)	• Genome wide loss-of-function screens performed to determine factors necessary for SVV-001 infection and replication	• ANTXR1/TEM8 was necessary for SVV-001 infection in neuroendocrine cancer cell lines
		• In neuroendocrine cancer cell lines, genetic knock out of ANTXR1/TEM8 was shown to drive loss of SVV-001 permissivity
		• Defective innate immune response was associated with SVV-001 replication
Hallenbeck and Chada (2021)	• Evaluation of efficacy of SVV-001 intratumoral injection combined with anti-PD-1 and anti- CTLA4 checkpoint blockade in an immunocompetent syngeneic pancreatic cancer murine model	• Combination treatment with intratumoral SVV-001 injection with anti-PD-1 and anti- CTLA4 checkpoint blockade led to both significant tumor shrinkage and improved survival

Although SVV-001 was found to target SCLC-N, the details of this interaction are more complex. The specific receptor of SVV-001 was recently discovered when Miles et al. (2017) performed genome wide loss of function screens and identified anthrax toxin receptor 1 (ANTXR1), also known as tumor endothelial marker 8 (TEM8), as the receptor for SVV-001 on tumor cells. The authors also established that TEM8/ANTXR1 expression alone was not sufficient for infective permissibility, and that decreased expression of antiviral IFN genes must also be present. Again, this group confirmed the association with SCLC-N, when they evaluated neurogenic transcription factors in responders and non-responders and also found that the elevated *NEUROD1* and low *ASCL1,* markers of SCLC-N, were associated with downregulation of antiviral IFN gene signaling (Miles et al., 2017). The same group also established that glycosylation of the TEM8/ANTXR1 receptor was necessary for SVV-001 binding, cell entry, and infection (Jayawardena et al., 2021). Although this association was identified in SCLC, it is likely, given TEM8/ANTXR1 is the receptor for SVV-001, that SVV-001 permissive subtypes of other neuroendocrine cancers share similar features to SCLC-N, including elevated TEM8/ANTXR1 and low expression of IFN genes.

## TEM8/ANTXR1: A marker of hypoxia, vasculogenic mimicry, and mediator of metastasis

**FIGURE 1:**
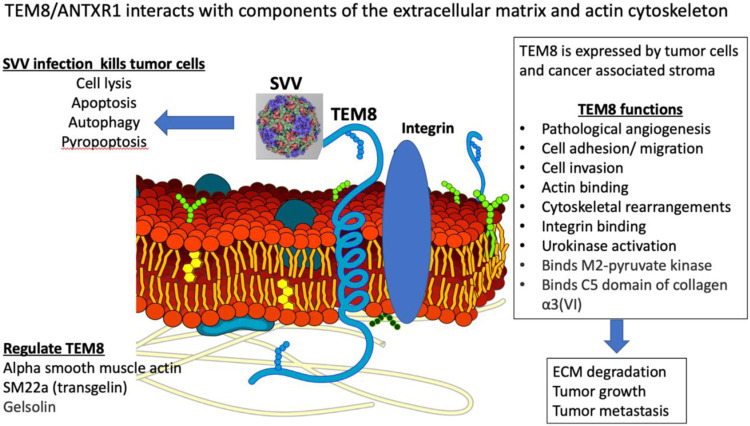
Illustration describing TEM8/ANTXR1, its function and anti-tumor effects.

TEM8/ANTXR1 is an integrin-like, transmembrane glycoprotein upregulated in a variety of cancer types, tumor associated stromal cells, and tumor-associated blood vessels (Yang et al., 2011; Evans et al., 2018) (Figure 1). TEM8/ANTXR1 is upregulated in the presence of hypoxia (Opoku-Darko et al., 2007). TEM8/ANTXR1 is unique in its association with tumor vessels but not normal blood vessels (Chaudhary et al., 2012). TEM8/ANTXR1 has been described as a marker for pathological, tumor-associated angiogenesis, which promotes tumor growth and may mediate resistance to therapies targeting angiogenesis (Xu et al., 2021).

Studies have demonstrated that TEM8/ANTXR1 is enriched in triple negative breast cancer (Xu et al., 2021), prostate cancer (Li et al., 2021a), gastric cancer (Li et al., 2021b; Sun et al., 2021), pancreatic cancer (Alcalá et al., 2019), angiosarcoma (Kusaba et al., 2021), colon cancer (Ł et al., 2021), and non-small cell lung cancer (NSCLC) (Gong et al., 2021). In multiple tumor types, upregulation of TEM8/ANTXR1 is a negative prognostic indicator (Li et al., 2021a; Ł et al., 2021; Ding et al., 2021). In triple negative breast cancer, TEM8/ANTXR1, is marker of vasculogenic mimicry, and is associated with poor outcomes (Fernández-Cortés et al., 2019; Xu et al., 2021). Vasculogenic mimicry is a process where tumor cells organize themselves into structures mimicking endothelial cells with functional tubes that can carry red blood cells. This process is driven by hypoxia. The presence of vasculogenic mimicry is associated with poor prognosis of multiple cancer types (Fernández-Cortés et al., 2019; Wei et al., 2021). Early evidence suggests that vasculogenic mimicry is mediated by tumor associated macrophages (Barnett et al., 2016; Rong et al., 2016; He et al., 2021). In addition, overexpression of TEM8/ANTXR1 in the setting of hypoxic tumor microenvironments is associated with the presence of cancer stem cells, increased stem cell self-renewal and increased metastasis in a Wnt pathway dependent mechanism (Chen et al., 2013). The interplay between cancer stem cells, TEM8/ANTXR1, angiogenesis, and tumor associated macrophages is a potentially important area for further studies.

TEM8/ANTXR1 is an adhesion molecule and meditates cell movement by binding to components of the extracellular matrix (ECM) and interacting with the actin cytoskeleton (Hotchkiss et al., 2005; Abdel-Hamid et al., 2019). The specific interaction between TEM8/ANTXR1 and the surrounding cells that mediates increased metastatic potential is not fully understood. TEM8/ANTXR1 interacts with the alpha 3 subunit of collagen VI which has been hypothesized to mediate cell attachment to endothelial cells and influence angiogenesis (Nanda et al., 2004; Hotchkiss et al., 2005; Werner et al., 2006). Although capillary morphogenesis protein 2 (CMG2) or anthrax toxin receptor 2 (ANTRX2) is the main mediator of anthrax toxicity (Liu et al., 2013a), TEM8/ANTRX1 was first identified as another target of anthrax toxin binding, specifically a site of binding of the protective antigen (PA) component of the anthrax toxin. TEM8/ANTRX1 contains a von-Willebrand factor A (vWA) domain that is involved in binding of PA (Bann, 2012). Low density lipoprotein receptor-related protein 6 (LRP6) has also been identified as an important component of the interaction of PA with TEM8/ANTRX1 in a process that also involves the Wnt/β-catenin signaling pathway (Wei et al., 2006; Peröbner et al., 2012). Other studies have also shown a connection between TEM8/ANTXR1 and endothelial cell response to Wnt signaling in cancer, with upregulation of TEM8/ANTXR1 associated with activation of downstream targets of Wnt pathways (Verma et al., 2011). In NSCLC cell lines, TEM8/ANTXR1 promotes metastasis *via* activation of Wnt/β-catenin signaling pathway (Ding et al., 2021). In hepatocellular carcinoma (HCC) cell lines microRNA-493 suppressed tumor cell growth by targeting TEM8/ANTXR1 and R-Spondin 2 (RSPO2) and decreasing activation of the Wnt/β-catenin signaling pathway (Xu et al., 2017). In glioblastomas upregulated TEM8/ANTXR1 is also a negative prognostic factor. Specifically, in a recent preprint, upregulation of hypomethylated TEM8/ANTXR1 genes in glioblastomas is associated with increased proliferation, metastasis, and resistance to chemotherapy and radiotherapy. The authors suggest that TEM8/ANTXR1 upregulation leads to β-catenin induction in a non-Wnt ligand dependent process (Kundu et al., 2022). Additional research is needed to fully clarify the role of TEM8/ANTXR1 in activation of Wnt/β-catenin signaling pathways and the relationship between this pathway and outcomes in different cancer types.

Studies with agents directly targeting TEM8/ANTXR1 have shown promising responses. One study showed genetic disruption of TEM8/ANTXR1 in a variety of human tumor xenograft models including melanoma, breast, colon, and lung cancer led to decreased tumor growth. In addition antibodies against TEM8/ANTXR1 have demonstrated anti-tumor activity and had synergistic effects with other anti-cancer agents (Reddy et al., 2007; Chaudhary et al., 2012). TEM8/ANTXR1 has been developed as a target in CAR-T therapy in breast cancer (Byrd et al., 2018; Petrovic et al., 2019). In preclinical murine models, an antibody-drug conjugate targeting TEM8/ANTXR1 led to tumor regression and improved survival (Szot et al., 2018). Antibodies blocking the TEM8/ANTXR1 extracellular domain inhibit tumor related angiogenesis and tumor growth (Opoku-Darko et al., 2007; Chaudhary et al., 2012; Gong et al., 2018; Szot et al., 2018). Studies are also evaluating immune-PET imaging agents to identify TEM8/ANTXR1 expression using a radiolabeled monoclonal antibody (Kuo et al., 2014). TEM8/ANTXR1 is a promising biomarker to select patients who may benefit from SVV-001 therapy, and additionally, there may be a role for combination therapy with additional agents that also target TEM8/ANTXR1 and associated pathways.

Although the receptor for SVV-001 has been identified, the role of a type 1 IFN response in SVV-001 efficacy as an OV remains to be fully clarified. Stimulator of interferon genes (STING) plays a major role in mediating type 1 interferon immune responses in viruses and cancer (Jiang et al., 2020). SCLC-N, known to be permissive to SVV-001, has decreased STING induced cytokines as compared to other SCLC subtypes, including reduced CCL5 and CXCL10 as described in the supplementary materials to the recent paper by Gay et al. (2021). In addition to host factors leading to decreased type 1 IFN signaling, SVV-001 itself seems to target local IFN host signaling response. SVV-001 inhibits type 1 IFN response when a SVV-001 associated protease, 3C protease, cleaves mitochondrial antiviral signaling (MAVS), Toll/interleukin 1 (IL-1) receptor domain-containing adaptor inducing IFN-β (TRIF), and TRAF family member-associated NF-κB activator (TANK) leading to loss of pattern recognition receptor (PRR) activation and decreased IFN production (Qian et al., 2017). In addition, SVV-001 has significant deubiquitinating activity which also contributes to the SVV-001’s ability to escape innate immune responses (Xue et al., 2018). SVV-001 replication also has been shown to induce degradation of retinoic acid-inducible gene I (RIG-I) a cytoplasmic PRR involved in type 1 IFN response which likely further contributes to decreased IFN production in SVV-001 infection (Wen et al., 2019). Finally, SVV-001 was found to kill tumor cells by inducing apoptosis in a process that involves SVV-001 proteins 2C and 3C protease and activation of caspase 3. This included mechanisms of apoptosis triggering both extrinsic death receptor signaling and intrinsic mitochondrial signaling pathways (Liu et al., 2019). This is particularly important as activation of capase-3 is associated with immunogenic cell death which is a critical component of OV efficacy in the development of anti-tumor immune response (Jaime-Sanchez et al., 2020). Interestingly, in pigs the mechanism of SVV-001 induced cell death differs from humans with induction of pyroptosis, a form of necrotic regulated cell death (Tsuchiya, 2021). In pigs SVV-001 3C protease cleaves porcine gasdermin D inducing pyroptosis (Wen et al., 2021). Taken together this data suggests a process where SVV-001 exploits a cellular environment with low expression of Type 1 IFN response to infect tumors and may also act to contribute this state, however, then infects and destroys the tumor cells leading to immunogenic cell death, which, when used as an OV has the potential synergize with checkpoint blockade to destroy tumors.

## Seneca valley virus studies in mice and humans

The initial preclinical and clinical studies of SVV-1 were completed in the 2000s before the current (2017) understanding of the role of TEM8/ANTXR1 as the receptor for SVV-001 had been developed. However, preclinical data for use as an OV in human tumors with neuroendocrine features was extraordinarily promising. In early mouse models of both SCLC and pediatric retinoblastoma a single dose of SVV-001 virus had remarkable efficacy with rapid killing of neuroendocrine tumor cells and minimal toxicity (Reddy et al., 2007). SVV-001 was also evaluated in a murine model of metastatic retinoblastoma and demonstrated that systemic injections of SVV-001 reduced the development of invasive disease as well as reduced central nervous system (CNS) metastatic lesions (Wadhwa et al., 2007). Another study evaluated the efficacy of SVV-001 *in vitro* in 23 cell lines including neuroblastoma, Ewing, sarcoma, and rhabdomyosarcoma panels. SVV-001 demonstrated high efficacy in both *in vivo* and *in vitro* murine models with objective responses most notably in rhabdomyosarcoma and neuroblastoma models (Morton et al., 2010). SVV-001 was evaluated in a murine model of pediatric malignant gliomas and a single injection of SVV-001 led to infection of xenografts without harming normal brain cells. This study also demonstrated efficacy and prolonged survival in permissive mouse tumor models (Liu et al., 2013b). Nonetheless, nearly all of the preclinical *in vitro* studies done with SVV-001 in murine models were somewhat limited as they were done in immunodeficient mice and the behavior of SVV-001 in immunocompetent models was not well defined.

Given the excellent preclinical data suggesting safety and efficacy in mouse models, phase 1 trials of systemic administration of SVV-001 were developed for both adults and children (Table 2). The first trial was a phase 1 dose escalation study in adults with advanced solid tumors with neuroendocrine features. Five cohorts were evaluated with a single intravenous dose of SVV-001 increasing in log increments from 10^7^ to 10^11^ viral particles/kg. The primary objectives were assessment of toxicity and determination of recommended dose. Secondary endpoints included serial assessment of viral titers in body fluids and blood and of neutralizing antibody titers. Systemic infusion of SVV-001 was well tolerated, however, several patients in the lowest dose cohort developing flu like symptoms within the first week. In the SCLC patients’ viral titers peaked at day 3–4 suggesting a delay in viral clearance, possibly explained by SVV-001 production within cancer cells. Response was evaluated and revealed 1 SCLC patient with rapidly progressive, extensive disease whose disease became stable after SVV-001 treatment with stability that persisted for >10 months. In five other patients with neuroendocrine tumors responses were noted; one patient with a carcinoid tumor had a 50% decrease in tumor size after SVV-001 administration (Rudin et al., 2011).

**TABLE 2:** Human clinical trials of SVV-001.

References	Study description	Outcomes
Rudin et al. (2011)	Phase 1 dose escalation trial of systemic SVV-001 in adults with advanced cancers with neuroendocrine differentiation (*N* = 30). Primary objectives were toxicity assessment and determination of recommended dose. Secondary objectives included assessment of viral titers and neutralizing antibody titers	SVV-001 was well tolerated with no dose limiting toxicities up to 10^11^ vp/kg. Intratumoral viral replication was detected as well as evidence of disease response in a patient with SCLC. All patients developed neutralizing antibodies
Burke et al. (2015)	Phase 1 dose escalation trial of systemic SVV-001 in children with advanced cancers with neuroendocrine differentiation (*N* = 22). Primary objectives were determination of maximum tolerated dose for SVV-001 as a single infusion (cohort A) or as two consecutive infusions in combination with cyclophosphamide (Cohort B). Secondary objectives included assessment of viral titers and neutralizing antibody titers	SVV-001 was well tolerated, one patient experienced a dose limiting toxicity in Cohort A (pain successfully treated with analgesics). No objective responses were observed. Neutralizing antibodies developed in both cohorts
Schenk et al. (2020)	Phase 2 double blind, placebo controlled trial of systemic SVV-001 in adults with extensive stage SCLC after first line chemotherapy. Primary endpoint was progression free survival (PFS). Secondary objectives include overall survival, response, and presence of neutralizing antibodies and viral clearance	Systemic SVV-001 was not associated with significant change in median PFS. In the SVV-001 group patients who had persistent detection of SVV-001 in peripheral blood 7 or 14 days after treatment had shorter PFS.

A phase 1 dose escalation trial was also done of systemic injection of SVV-001 in children with advanced neuroblastoma, rhabdomyosarcoma, or tumors with neuroendocrine features. The trial had 2 cohorts, cohort A was a dose escalation group with 3 increasing dose levels. In Cohort B patients were treated with two doses of SVV-001 given at day 8 and day 29 in combination with oral cyclophosphamide to modulate immune antiviral response. In total, 22 patients were enrolled on the study. No patients had objective responses, 6 of 12 evaluable patients in part A and 4 of 6 evaluable patients on part B had stable disease. All patients in part A cleared SVV-001 from their blood within 3 weeks of treatment. In part B viral titers were cleared within 2 weeks of infusion. Neutralizing antibodies were present in all patients (Burke et al., 2015).

A phase II randomized, placebo controlled study with systemic SVV-001 versus placebo was done in adults with extensive stage SCLC with disease that was either stable or responding after at least 4 cycles of platinum based chemotherapy. The primary endpoint of this study was progression free survival. In this trial 59 patients were randomized to receive SVV-001 versus placebo. Efficacy was assessed at a prespecified interim futility analysis after 40 events. This interim analysis did not demonstrate efficacy with median progression free survival (PFS) of 1.7 months in both study and placebo arms. No significant overall survival (OS) difference was observed. Neutralizing antibodies were detected at 2 weeks in all patients tested, and viral clearance was noted in majority of patients by 14 days after treatment. There were very few patients who had persistent viral titers. Persistent viral titers were attributed to intratumoral replication of SVV-001. Exploratory analysis was performed and delayed clearance of virus was associated with decreased PFS (Schenk et al., 2020). This is now thought to be due to selective viral replication in patients with TEM8/ANTRX1 enriched tumors, which confers poor prognosis in various tumor types.

OVs can be delivered systemically or with direct intra-lesional injection into tumors (Zheng et al., 2019; Cook and Chauhan, 2020). The advantage of systemic administration include ease of administration and improved targeting of metastatic disease (Atasheva and Shayakhmetov, 2021). Prior studies in neuroendocrine cancer models with other OV therapies have demonstrated success with systemic infusions of OVs in combination with other immunomodulatory agents (Inoue et al., 2022). Disadvantages of systemic injection include development of antiviral neutralizing antibodies and cytotoxic T lymphocytes and possible off target adverse effects. The anti-viral immune response likely limits both intratumoral viral infection and anti-tumor efficacy of OVs. The only FDA approved OV, T-VEC, is delivered by intratumoral injection (Andtbacka et al., 2015). Intratumoral injection overcomes the barriers to efficacy from the development of neutralizing antibodies, but makes delivery of the OV more difficult for patients with inaccessible sites of disease.

**FIGURE 2:**
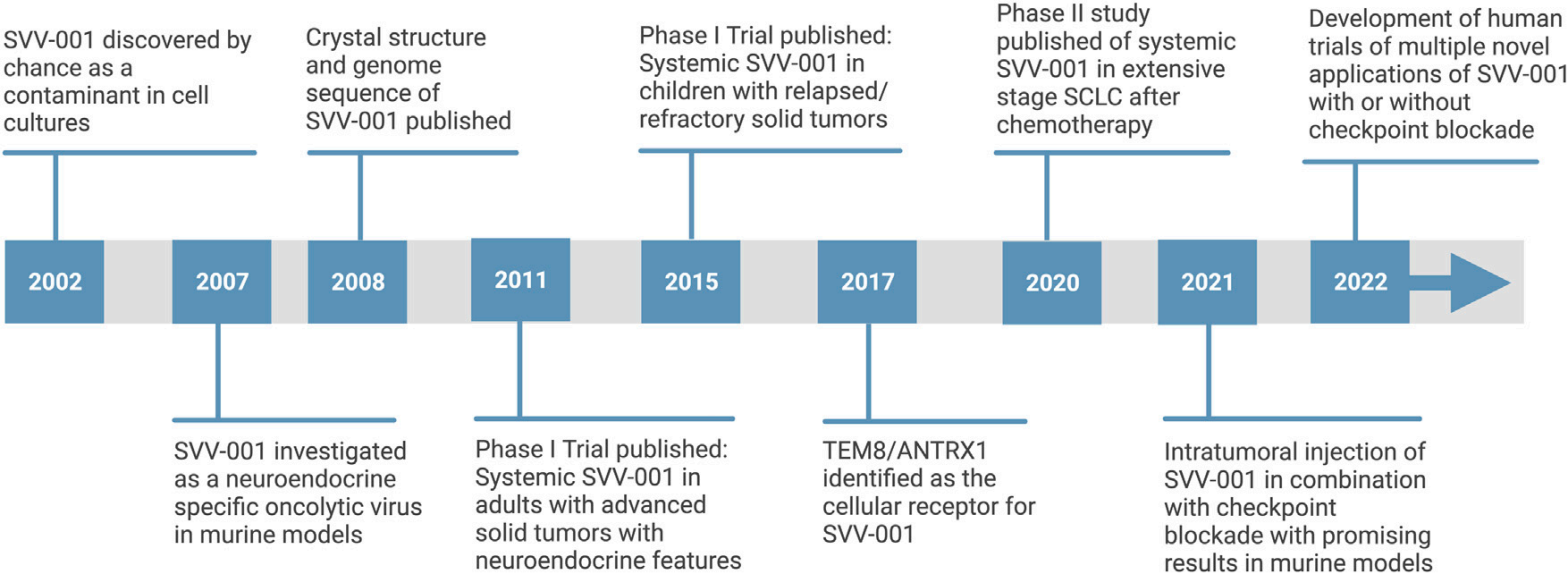
Timeline of SVV-001 oncolytic virus development [adapted from “timeline (7 segments, horizontal),”by BioRender.com (2022). Retrieved from https://app.biorender.com/biorender-templates].

**FIGURE 3:**
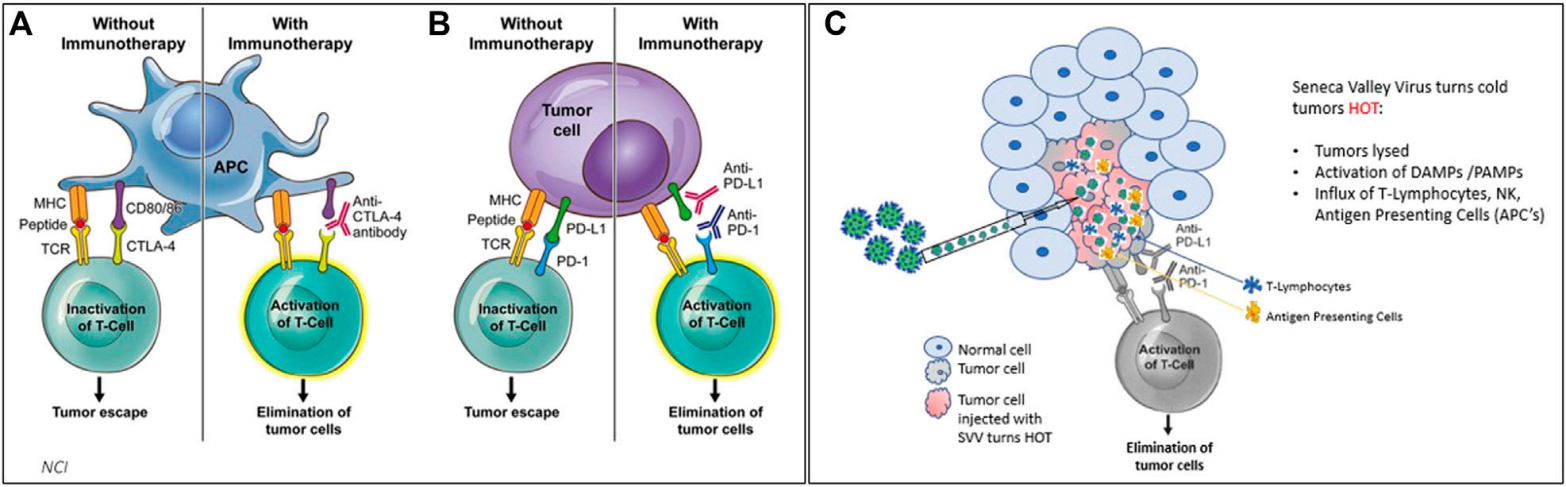
**(3A,B)** Treating cancer with checkpoint inhibitors (CPI’s) achieves responses in solid cancers that are defined as “hot” tumors (with immune cells such as APCs and T cells) ∼25% response rate on average observed. **(C)** when SVV is administered either systemically or intratumorally, SVV makes tumors HOT. SVV also replicates inside of the tumor, causing an immune response which activates DAMPs and PAMPs, and creates an influx of T-cells, NK cells, and antigen presenting cells to attack tumor cells.

Multiple early clinical trials showed that SVV-001 is safe with systemic administration (Figure 2). In these three studies, SVV-001 was administered in 1 or 2 IV infusions to a total of 76 patients at doses up to 10^11^ vp/kg. About 49 of these patients received highest dose with just one observed DLT. This DLT was tumor pain, which was successfully treated with analgesics. Although these studies did not show significant response with a systemic administration of SVV-001 as a monotherapy, subgroups of patients, did signal response. As stated previously, all clinical studies were done prior to the discovery that TEM8/ANTXR1 is the receptor for SVV-001 on tumor and stromal cells and a potentially valuable biomarker for patients who would most benefit from therapy with SVV-001. In addition, rapid technological advancement in the study of OVs has shown that intratumoral injection of OVs has the potential to deliver local impact as well as distant abscopal responses and may represent a more effective means of targeting tumors than systemic administration (Melero et al., 2021). Rational combinations of intratumor administration of OVs in combination with checkpoint blockade has a great potential for synergy as OVs induce immunogenic cell death by activating both innate and adaptive immune responses can potentially enhance the efficacy of checkpoint blockade, Figure 3 (Workenhe and Mossman, 2014; Ma et al., 2020; Boagni et al., 2021; Zhou et al., 2021).

The specific proposed mechanism of combination of checkpoint blockade with OV includes modulation of an immune excluded microenvironment to enhance activity of cytotoxic T cells. Neuroendocrine cancers including well differentiated neuroendocrine tumors and SCLC-N do not respond to checkpoint blockade (Takkenkamp et al., 2020; Gay et al., 2021) which is thought to be mediated by an immune excluded tumor microenvironment. The exact mechanism of this is not clear, in SCLC-N, this may be due to evasion of natural killer surveillance (Zhu et al., 2021), however, in several types of neuroendocrine cancers tumor associated macrophages likely also play a role (Cai et al., 2019). To overcome the immune suppressive and tumor permissive environment, OV therapy with SVV-001 triggers immunogenic cell death after injection into TEM8/ANTXR1 enriched tumors cells and the associated TEM8/ANTXR1 enriched stromal cells. This leads to lysis of tumor cells and stromal cells and triggers release of DAMPs which draw innate immune cells including dendritic cells, key activators of tumor specific T cells and response to checkpoint blockade, to the microenviroment (van Vloten et al., 2018). In addition, release of tumor antigens further primes immune responses and promotes tumor infiltrating lymphocyte recruitment (Harrington et al., 2019). Lastly, RNA from both SVV and lysed cells triggers DAMPs and PAMPs to accentuate immune response. Overall, these processes enhances the efficacy of checkpoint blockade to overcome the cancer permissive and immune excluded microenvironment.

In addition OVs can be engineered to deliver cytokines to the tumor microenvironment in combination with checkpoint blockade (Nakao et al., 2020). Early studies suggest efficacy of OVs combined with CAR-T cells therapy (Rezaei et al., 2021; Rosewell Shaw et al., 2021) and bispecific antibodies (Heidbuechel and Engeland, 2021). As our understanding of the tumor microenvironment unfolds, genetically engineered OVs will allow precise manipulation of the tumor microenvironment alone or in combination with other immunotherapy agents. Given the rapid advances in immunology in the last 5 years and the discovery of a specific biomarker for SVV-001, the next generation of SVV-001 based therapies is being developed.

Studies in murine models using SVV-001 in combination with checkpoint blockade are already very promising. One study evaluated intratumoral injection of SVV-001 in combination with checkpoint blockade in two murine models of neuroblastoma and melanoma engineered with upregulated TEM8/ANTXR1 receptors. In this study both cell lines were resistant to checkpoint blockade at baseline. The combination of checkpoint blockade plus SVV-001 increased the response rate up to 6-fold over checkpoint inhibition alone (*p* < 0.01) (Hallenbeck and Chada, 2021). Finally, a phase I/II trial is already in development exploring SVV-001 administered intratumorally in combination with ipilimumab and nivolumab compared to ipilimumab and nivolumab alone in TEM8/ANTXR1 enriched neuroendocrine tumors and neuroendocrine carcinomas (Wire, 2021a). This novel study is based on preclinical data from Seneca Therapeutics, Inc. SVV-001 was injected intratumorally in a pancreatic cancer model (Pan02) in combination with anti PD1and/or anti CTLA4 antibodies. SVV-001 not only re-sensitized tumors to immune checkpoint inhibitors but also resulted in synergistic antitumor activity as compared to immune checkpoint inhibitors alone. Over 83% of mice were noted to have compete responses with combination SVV-001 plus both immune checkpoint inhibitors. Responses were not only noted in injected lesions but also when the mice were challenged with naïve pan02 cells on the contralateral flank. Only mice from animals that had tumors regress from treatment with SVV-001 plus anti PD1 and anti CTLA4 antibodies rejected the challenge, suggesting a systemic abscopal effect. It is well known that OVs induce T-cell infiltration in injected tumors. This was also noted in SVV-001 preclinical investigations with the combination of SVV-001 and immune checkpoint demonstrating the highest T cell infiltration. Interestingly, tumors regressed with multiple injections of SVV plus CPIs despite the presence of high concentrations of SVV neutralizing antibodies, again suggesting that antibodies aren’t effective in blocking SVV when injected at high concentrations inside a tumor. These data were presented at the 2022 AACR symposium (Hallenbeck and Chada, 2021).

Seneca Therapeutics has created a novel 8 gene reverse transcription polymerase chain reaction (RT-PCR) assay, performed on formalin fixed paraffin embedded patient tumor samples commonly available from most solid cancer patients. This test detects TEM8/ANTXR1 as well as seven additional genes to accurately predict if the patient’s tumor is permissive to SVV infection. This test will be used to screen potential patients intended for SVV-001 therapy in clinical trials (Wire, 2021b). In addition, the development of a cancer gene delivery platform is underway allowing the incorporation of immunomodulatory transgenes into a SVV-001 delivery system allowing precise targeting of the tumor immune microenvironment of TEM8/ANTXR1 enriched tumors (Wire, 2021c).

## Seneca valley virus in small cell lung cancer

SCLC is an aggressive cancer in dire need of effective treatments. The potential for SVV-001 in combination with checkpoint blockade to target SCLC has been further informed by recent advances in understanding of the pathophysiology of SCLC. Specifically, greater understanding of the SCLC molecular subgroup SCLC-N, with elevated *NEUROD1* and low *ASCL1*, targeted by SVV-001 shed light on the mechanisms of viral entry and efficacy as well as possible future targets for SVV-001-derived therapies. Rudin et al. describe four subtypes of SCLC based on expression of transcription regulators including SCLC-A, defined as *ASCL1*-high, SCLC-N, defined as *NEUROD1*-high, SCLC-Y defined as *YAP1* high, and SCLC-P defined as *POU2F3* high (Schwendenwein et al., 2021). In addition evidence from murine models suggest that *ASCL1* rather than *NEUROD1* is key to tumorigenesis of SCLC (Borromeo et al., 2016) and that over time c-MYC enriched tumor cells arise in this population and drive a switch to a *NEUROD1* high state. In mouse models MYC driven, *NEUROD1* high tumors are sensitive to Aurora kinase inhibition (Mollaoglu et al., 2017). This finding was further explored in a phase II, randomized, placebo-controlled trial of paclitaxel plus alisertib (an Aurora kinase inhibitor) as second line treatment in SCLC, with a primary endpoint of PFS. Although PFS was not significantly improved in an unselected patient population, in exploratory studies c-Myc expression by immunohistochemistry (IHC) was associated with improved PFS (4.64 months in paclitaxel/alisertib versus 2.27 months paclitaxel/placebo) (Owonikoko et al., 2020). In other models c-MYC was associated with transition from SCLC-A to SCLC-N and also regulation of Notch signaling pathways involved in epithelial-to-mesenchymal transition (Patel et al., 2021a). Whether aurora kinase inhibition could have synergy with SVV-001 is an open area of investigation.

In another recent paper by Chan et al. (2021) plasticity and immunosuppression in SCLC was explored in both primary tumors and metastases through single cell transcriptome sequencing and imaging techniques. They noted that SCLC-N was enriched in metastasis while primary tumors were more commonly SCLC-A. In addition, SCLC-N were found to express lower levels of immune-related genes as compared to SCLC-A, suggesting an immune “cold” tumor microenvironment. Consistent with this, SCLC-N was associated with T cell dysfunction including higher levels of Treg cells and CD8 + exhausted phenotype, with evidence of reduced cytotoxic CD8^+^ effector cells. SCLC-N was also associated with increased markers of epithelial-mesenchymal transition, transforming growth factor-β (TGF-β), and other markers of pro-metastatic gene expression. Finally, SCLC-N cells were associated with a pro-fibrotic and immunosuppressive population of monocytes and macrophages.

Interestingly, somatostatin receptor 2 (SSTR2) upregulation is also associated with *NEUROD1* expression in both SCLC cell lines and primary tumors, and correlates with worse clinical outcomes (Lehman et al., 2019; Gay et al., 2021). SSTR2 is an important target in well differentiated neuroendocrine tumors which have high expression of this receptor (Caplin et al., 2014). However, targeting of SSTR2 in high grade neuroendocrine carcinoma has not shown significant responses (Macaulay et al., 1991; Lapa et al., 2016). Given that SVV-001 targets both well differentiated and high-grade neuroendocrine tumor, and that SSTR2 may be upregulated in the same tumors, one might hypothesize that SSTR2 may play a role in a specific type of tumor microenvironment characterized by upregulated TEM8/ANTXR1, low expression of type 1 IFN associated genes, immunosuppressive myeloid infiltration, and pathological tumor associated angiogenesis. In older studies SSTR2s are upregulated in neo-angiogenesis (Curtis et al., 2000; Watson et al., 2001; Adams et al., 2005). Synergy between SSTR2 directed therapies and SVV-001 could be evaluated in future studies.

IMpower 133, a clinical trial of chemotherapy in combination with immune checkpoint blockade, was a major breakthrough in SCLC, long thought to be recalcitrant to immunotherapy based treatment regimens (Horn et al., 2018). However, this study was done in an unselected patient population and as the current understanding of SCLC pathophysiology has developed with a focus on SCLC subgroups, biomarker driven studies represent an important advancement in therapeutic trial development for SCLC. Gay et al. (2021) confirmed this paradigm in their recent exploration of SCLC treatment response in IMpower 133 classified by transcriptomic subgroups. They describe an emerging new group, SCLC-I or an inflamed SCLC subgroup, more likely to respond to checkpoint blockade. Within the population of treatment naïve patients enrolled, 17% of patients were SCLC-I and 23% of patients were found to be SCLC-N (Gay et al., 2021). The upcoming phase I/II clinical trial of intratumoral SVV-001 in combination with ipilimumab and nivolumab represents the next generation of truly biomarker-driven drug development for SCLC with selection of patients based on TEM8/ANTXR1 expression. Although SCLC-Ns are thought to be “cold” tumors poorly responsive to checkpoint blockade, with the addition of SVV-001, this trial promises to bring the advances of immunotherapy to patients with this aggressive and highly morbid disease.

## Seneca valley virus in extra-pulmonary high-grade neuroendocrine carcinoma

Although SCLC is the most well-known high-grade neuroendocrine carcinoma, extra-pulmonary high-grade neuroendocrine carcinoma is also associated with significant mortality. High grade extra-pulmonary neuroendocrine carcinomas can arise throughout the body, are similar to SCLC in that they are aggressive tumors causing limited life expectancy (Dasari et al., 2018; McNamara et al., 2020). Although not as well defined as in SCLC, recent studies have also explored transcriptomic subgroups in extra-pulmonary high grade neuroendocrine carcinoma and revealed transcriptomic subgroups defined by expression of *NEUROD1* and *ASCL1* (Kawasaki et al., 2020; Li et al., 2021c; Metovic et al., 2022). However, data is limited given the rarity of these tumors, and there are no clearly defined transcriptomic subgroups as in SCLC that may predict response to checkpoint blockade in high grade extra pulmonary neuroendocrine carcinoma. Microsatellite instability and elevated tumor mutation burden (TMB) > 10 may predict response to checkpoint blockade in this setting (Sahnane et al., 2015; Girardi et al., 2017; Shao et al., 2020). First line treatment in extra pulmonary high grade neuroendocrine carcinoma is combination of platinum and etoposide (Thomas et al., 2019). The use of immunotherapy was explored in the phase II Dual Anti-CTLA-4 and Anti-PD-1 Blockade in Rare Tumors (DART) SWOG S1609 trial which reported a 26% overall response rate with ipilimumab and nivolumab in patients with high grade extra-pulmonary neuroendocrine neoplasms. In subgroup analysis of this trial there were several responders with microsatellite stable disease and TMB < 10 (Patel et al., 2021b). Further biomarkers of response to immunotherapy are needed. SVV-001 represents a promising agent in this setting, as above, with the ability to provide biomarker driven therapy. The planned phase I/II trial will include all neuroendocrine carcinomas and promises to deliver not only responses in this aggressive disease, but also an expanded understanding of these rare but aggressive cancers with help of serial tumor biopsies and exploratory correlative studies.

## Seneca valley virus in well differentiated neuroendocrine tumors

Neuroendocrine tumors are distinct from neuroendocrine carcinomas in their relatively indolent disease course and characteristic morphology microscopically. Neuroendocrine tumors can originate from anywhere in the body but small intestine, lung, and pancreas constitute the most prevalent locations. The WHO classification of both pulmonary neuroendocrine neoplasms and gastroenteropancreatic (GEP) neuroendocrine neoplasms were recently updated (Assarzadegan and Montgomery, 2020; Nagtegaal et al., 2020; Nicholson et al., 2022). The incidence of well differentiated neuroendocrine tumors is increasing (Dasari et al., 2017). There are limited FDA approved therapies for oncologic treatment of neuroendocrine tumors; these include lanreotide (Caplin et al., 2014), everolimus (Yao et al., 2011; Yao et al., 2016), and Peptide Receptor Radionuclide Therapy (PRRT) (Strosberg et al., 2017). Promising studies of multi-target tyrosine inhibitors are ongoing (Chan et al., 2017; Capdevila et al., 2018; Grillo et al., 2018; Capdevila et al., 2021). Prior studies have evaluated checkpoint blockade in well differentiated neuroendocrine tumors with limited overall response rates (Mehnert et al., 2020; Strosberg et al., 2020; Yao et al., 2021).

Well differentiated neuroendocrine tumors were known to be permissive to SVV-001. Although there is no published data about TEM8/ANTXR1 upregulation in well differentiated neuroendocrine tumors, given the permissivity towards SVV-001, it is likely that upregulated TEM8/ANTXR1 is present in a subset of these tumors. It is clear that well differentiated neuroendocrine tumors express high levels of SSTR2 (Wolin, 2012), but a connection between SSTR2 and pathologic angiogenesis, possibly associated with TEM8/ANTXR1 is only speculation at present.

The same transcriptomic subgroups explored in SCLC and more recently in extra-pulmonary high-grade neuroendocrine carcinoma have not been defined in well differentiated neuroendocrine tumors. Past data suggests that subsets of well differentiated gastroenteropancreatic neuroendocrine tumors do express elevated *NEUROD1* (Shida et al., 2008). One study examining small intestinal neuroendocrine tumors using transcriptomic expression profiling identified three clusters of small intestinal neuroendocrine tumors with different patient survival patterns. In 2 of the 3 clusters identified, *NEUROD1*, was found to be an upstream transcriptomic regulator (Andersson et al., 2016). In addition, well differentiated neuroendocrine tumors are known to be highly vascular, which is the basis of the “neuroendocrine paradox” where in contrast to adenocarcinomas, lower grade, more indolent tumors often have increased dense vascular networks as compared to higher grade more aggressive tumors (Scoazec, 2013; Carrasco et al., 2017). Well differentiated pancreatic neuroendocrine tumors are associated with hypoxia driven, abnormal angiogenesis, and vascular mimicry (Chu et al., 2013). Pancreatic neuroendocrine tumors are also associated with C-MYC overexpression which also promotes vascular endothelial growth factor C (VEGFC) expression the development of lymphatic endothelial cells (Chang et al., 2021). In addition, well differentiated neuroendocrine of the midgut are associated with an immunosuppressive (Busse et al., 2020) and intensely fibrotic tumor microenvironment with crosstalk between tumor cells and stromal cells, and upregulation of integrin signaling pathways (Laskaratos et al., 2021). All of these characteristics suggest a type of hypoxia-driven highly vascular tumor microenvironment similar to the environment that in other tumor types are enriched for TEM8/ANTXR1. However, the pathophysiology of the development of this type of environment is likely different in well differentiated neuroendocrine tumors as compared to high grade neuroendocrine carcinomas. Recent evidence suggests plasticity in SCLC, where tumors starts as SCLC-A and transition to SCLC-N over time with environmental pressure (Ireland et al., 2020; Chan et al., 2021). Well differentiated neuroendocrine tumors develop from neuroendocrine cells, which are physiologically involved in complex hormonal paracrine and autocrine processes and closely interact with the local tissue environment and vasculature. It is likely that intrinsic processes, related to neuroendocrine cell function drive the local tumor microenvironment as these cells transform to neuroendocrine tumors.

SVV-001 is a potentially transformational agent for well differentiated neuroendocrine tumors. SVV-001 intratumoral injection in combination with checkpoint blockade may lead to significant responses in patients with TEM8/ANTXR1 upregulation. Current FDA approved agents used in well differentiated neuroendocrine tumors are often cytostatic. SVV-001 and immune checkpoint combination holds the potential for significant cytoreduction based on impressive pre-clinical data. This is especially needed for patients with large, bulky symptomatic disease.

## Discussion: Seneca valley virus beyond neuroendocrine neoplasms

SVV-001 is an important potential therapeutic agent in many cancer types. However, understanding SVV-001 and the unique tumor microenvironment, represented by upregulation of TEM8/ANTXR1, that it targets, has the potential to provide additional clues about mechanisms of resistance to immunotherapy and chemotherapy in neuroendocrine neoplasms and other cancers. TEM8/ANTXR1 upregulation has been described in a variety of solid tumors types including triple negative breast cancer (Xu et al., 2021), prostate cancer (Li et al., 2021a), gastric cancer (Li et al., 2021b; Sun et al., 2021), pancreatic cancer (Alcalá et al., 2019), angiosarcoma (Kusaba et al., 2021), colon cancer (Ł et al., 2021), and NSCLC (Gong et al., 2021). Lineage plasticity with a transformation from adenocarcinomas to carcinomas with neuroendocrine differentiation has been described in a variety of solid tumors, including lung and prostate primary tumors (Farrell et al., 2017; Rubin et al., 2020; Ito et al., 2021). This transformation is often associated with the development of therapy resistance and portends poor outcomes for patients. The tropism of SVV-001 for neuroendocrine cancer, mediated by upregulated TEM8/ANTXR1 can inform this paradigm and opens the door for novel uses of SVV-001 to target these tumor types. The model of lineage plasticity described in SCLC, with SCLC-A transforming to SCLC-N mediated by MYC activation (Ireland et al., 2020), has similarities to the transformation of prostate cancer (Li et al., 2021a) and pancreatic cancer (Farrell et al., 2017). The identification of TEM8/ANTXR1 as a potential mediator of neuroendocrine transformation was most clearly shown in prostate cancer, where N-MYC was found to promote dysregulated angiogenesis and tumor progression *via* TEM8/ANTXR1 (Li et al., 2021a). The specific association between upregulated TEM8/ANTXR1, vasculogenic mimicry, and cancer stem cells, suggests the presence of a hypoxic tumor microenvironment with disordered angiogenesis, which promotes the survival and spread of cancer cells. It remains to be clarified if this same pathway of lineage plasticity is also present in other tumor types expressing TEM8/ANTXR1, however it is possible the same paradigm mediates metastasis and therapy resistance in subsets of triple negative breast cancer, gastric cancer, colon cancer, and NSCLC. Further research is needed to validate if these hypotheses prove true and if they represent additional targets for cancer therapy.

SVV-001 was first identified as neuroendocrine specific OV, with extraordinary potential to transform the landscape of neuroendocrine neoplasm treatments by inducing a significant response in a tumor type long thought to be resistant to immunotherapy. However, early studies were limited by lack of a biomarker to select SVV permissive patients. The identification of TEM8/ANTXR1 as the receptor for SVV-001, where SVV-001 can be administered *via* intratumoral injections, in a biomarker enriched patient population and in combination with dual checkpoint blockade to optimize responses has paved the way for the next generation of rationally designed clinical trials using SVV-001. Although this treatment paradigm was developed to target neuroendocrine neoplasms, recent advances in the understanding of lineage plasticity of neuroendocrine transformation in a variety of solid tumor types along with studies identifying widespread TEM8/ANTXR1 upregulation, suggest that SVV-001 has the potential to target many other tumor types that are particularly therapy-resistant and deadly. Further understanding of the precise immune tumor microenvironment associated with TEM8/ANTXR1 upregulation in high grade neuroendocrine carcinoma, well differentiated neuroendocrine tumors, and other associated tumor types is key to not only using SVV-001 to target these diseases, but also to developing other novel agents that could be used in combination with SVV-001.
